# Assessment of different regions of interest-based methods for [99mTc]Tc DAT-SPECT quantification using an anthropomorphic striatal phantom

**DOI:** 10.1186/s40658-022-00519-2

**Published:** 2022-12-28

**Authors:** Leonardo Alexandre-Santos, Ana Carolina Trevisan, Felipe Arriva Pitella, Vitor Tumas, Jose Henrique Silvah, Mery Kato, Eder Rezende de Moraes, Lauro Wichert-Ana

**Affiliations:** 1 Nuclear Medicine and PET/CT Section, Department of Medical Imaging, Hematology, and Clinical Oncology, University of São Paulo (USP), Ribeirão Preto, Brazil; 2 Interunits Bioengineering Postgraduation Program, São Carlos School of Engineering, University of São Paulo (USP), São Carlos, Brazil; 3 Department of Physics, Faculty of Philosophy, Sciences and Literature of Ribeirão Preto (FFCLRP), University of São Paulo (USP), Ribeirão Preto, Brazil; 4 Interunits Bioengineering Postgraduation Program, São Carlos School of Engineering, University of São Paulo (USP), Ribeirão Preto, Brazil; 5 The Center for Interdisciplinary Research on Applied Neurosciences (NAPNA), Center of Nuclear Medicine, University of São Paulo (USP), São Paulo, Brazil

**Keywords:** Dopamine transporter, SPECT, Brain, Quantification

## Abstract

**Background and aims:**

Molecular imaging of the dopamine transporters (DAT) provides valuable information about neurodegenerative diseases, such as Parkinson’s. This study assessed the accuracy and precision of DAT-SPECT quantification methods.

**Methods:**

Twenty-three DAT-SPECT images of a striatal phantom were acquired. The specific (caudate and putamen) and the non-specific (background activity) chambers were filled with [^99m^Tc]Tc. Different specific-to-non-specific activity ratios (10, 9, 8, 7, 6, 5, 4, 3 and 2 to 1) and the specific binding ratio (SBR) were calculated. Five methods using ROIs were assessed: (a) Manual ROIs on SPECT images; (b) TwoBox and (c) ThreeBox methods and Volume of Interest (VOI) using structural images; (d) MRI and (e) CT. Accuracy was evaluated by the concordance correlation coefficient (CCC) and precision by Pearson’s coefficient and linear regression.

**Results:**

The SBR quantified in the specific and striatal chambers resulted in a CCC increase with a decrease in the nominal values. For lower SBR, MRI and CT showed higher CCCs when caudate ($\hbox {CCC}_{\mathrm{MRI-CA}}$ = 0.89 e $\hbox {CCC}_{\mathrm{CT-CA}}$ = 0.84) and putamen ($\hbox {CCC}_{\mathrm{MRI-PU}}$ = 0.86 e $\hbox {CCC}_{\mathrm{CT-PU}}$ = 0.82) were evaluated. For striatal assessments, the TwoBox method was the most accurate ($\hbox {CCC}_{\mathrm{TWOBOX-ST}}$ = 0.95). High Pearson’s coefficients were found in the correlations between all methods.

**conclusions:**

All five methods showed high precision even when applied to images with different activities. MRI and CT were the most accurate for assessing the caudate or putamen. To assess the striatal chamber and in the absence of structural information, the TwoBox method is advisable.

**Supplementary Information:**

The online version contains supplementary material available at 10.1186/s40658-022-00519-2.

## Introduction

Parkinson’s disease (PD) is a condition associated with the degeneration of the dopaminergic nigrostriatal neurons and intracytoplasmic inclusions (i.e., Lewy Corpuscles) [1], resulting in dopamine (DA) depletion in the striatum (ST) [2]. A prevalence of 100–300 cases of PD per 100,000 people was reported [3]. PD has been diagnosed according with clinical criteria. Errors in the evaluation of early disease cases reaches 25% among professionals with limited experience [3]. Moreover, in the initial phase of the disease, a lack of motor symptoms possibly due to “neuronal reserves” or active compensatory mechanisms could be observed [4]. Consequently, early clinical detection of PD is difficult due to absence of symptoms and characteristic signs, such as motor deficits, until 60–70% of the dopaminergic neurons have already been lost [5]. Anatomical imaging techniques, such as Computed Tomography (CT) and Magnetic Resonance Imaging (MRI) often do not evidence specific abnormalities in early cases of PD [6]. Otherwise, molecular methods, such as Positron Emission Tomography (PET) and Single Photon Emission Computed Tomography (SPECT), are capable of detecting metabolic and neurochemical changes caused by PD, like a decreased density of the dopamine transporters (DAT) on dopaminergic neurons. To evaluate the characteristics of the different radiopharmaceuticals [7, 8], methodologies of quantification based in regions-of-interest (ROI) have been developed [9, 10]. These vary according with different parameters of acquisition and reconstruction of images, as well as with how ROIs are created, if by hand or using templates with standardized geometries [11, 12]. Relative quantification or semi-quantification is widely used in SPECT images of the striatum (ST), allowing the investigation of dopamine neurons with functioning synapses by using binding ratios. These are proportions between the number of counts collected within ROIs constructed on a specific striatal region and counts of a non-specific ROI, typically positioned in the cerebellum or the posterior portions of the occipital lobe [13]. Robust techniques involving other medical imaging modalities, capable of providing a better spatial resolution of the structures of interest and accuracy in the collected signal [14–16] are not available for SPECT. Moreover, few studies were carried out to evaluate the efficiency among different quantification methods of the dopaminergic integrity in the ST. This study aimed to assess the performance of five semi-quantitative methods used for DAT images through of the performance of accuracy and precision under controlled simulation conditions using an anthropomorphic striatal phantom.

## Methods

### Phantom design

**Fig. 1:**
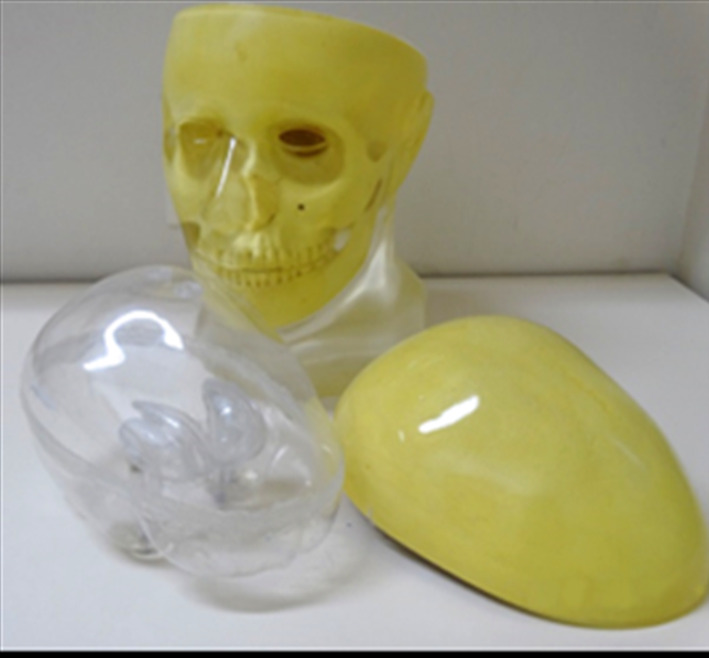
Striatal anthropomorphic phantom Alderson RSD and skull to attenuation correction in equivalent tissue

The striatal anthropomorphic phantom Alderson RSD (Radiological Support Devices, Long Beach, CA) was used for the SPECT images (Fig. 1). The phantom is composed of 4 compartments of interest with volumes and morphologies simulating the ST components with capacity of 4.7 ml and 4.6 ml for the caudate nucleus (CA) and 5.4 ml and 6.0 ml for the putamens (PU) on the right and left sides, respectively. These striatal compartments defined as volumes of interest were filled with the different known activity of $^{\textrm{99m}}$Tc. A fifth compartment defined as the brain shell was filled with a smaller activity than in the volumes of interest and used as a reference volume, representing a non-specific activity value (background counts) for the quantification processes. For the acquisition of images, the striatal phantom was attached to a support with tissue shape and density (0.23 g/$\hbox {cm}^{\textrm{3}}$) equivalent to the human skull. The $^{\textrm{99m}}$Tc activity concentration adopted was designed to simulate Binding Potential Index (BPI) similar to those observed clinically (0.8 to 4.0). For this, it was necessary to establish a ratio between the activities used to fill the compartments of interest and the brain shell. The different proportional ratios used in this study—10:1, 9:1, 8:1, 7:1, 6:1, 5:1, 4:1, 3:1, and 2:1—were separated into three activity level groups (high, intermediary and low) and 23 images were acquired simulating uptake patterns. All $^{\textrm{99m}}$Tc solutions were prepared using deionized water to avoid problems in the homogeneous distribution of technetium within each well of the phantom. At the end of each SPECT acquisition, 0.2 ml aliquots of each compartment were collected to measure activity values. The activity values recorded before and after the acquisition of images were used as correction parameters for the decay effects of the samples.

### Images acquisition and reconstruction

SPECT images were acquired in a gamma camera BrightView XCT (Philips Medical Systems Inc., Cleveland, OH, USA) consisting of two detector heads. The projection data were collected using a Low Energy High Resolution (LEHR) collimator and a 20 cm radius of rotation. In a step-shoot acquisition mode, 64 projections were acquired in a circular orbit of 180$^{\circ }$ per head and a 30s acquisition time by projection, a matrix of $128 \times 128$, the magnification factor of x1 on pixel dimension of 2.13 mm. The data were acquired from a symmetric energy window of width 20% and centered on 140 keV photopeak. The images were reconstructed using the 8-iteration and 4 subsets iteration algorithm (Ordered Subset Expectation Maximization (OSEM). The Chang’s method was used for corrections of the attenuation effects of gamma photons, using a linear attenuation coefficient of 0.11 $\hbox {cm}^{\mathrm{-1}}$, Butterworth filter of second order and cutoff frequency of 0.22 cycles/pixel. The MRI and CT images of the phantom were acquired by searching for the volumetric information of interest structures. For MRI, the 4 compartments of interest of the striatal simulator were carefully filled with a solution of 0.1 mM of $\hbox {CuSO}_{\textrm{4}}$, whereas only one volume of deionized water was used for the brain shell. A single T1-weighted MRI image with Echo Time (ET) of 3.35 ms, 9.7 ms Repetition Time (RT) was acquired on a Philips Achieva MRI device with a field strength of 3.0T (Philips Medical System, Best, The Netherlands). For CT, the brain shell was completely filled with only deionized water while the other compartments of the striatum were kept empty. The CT image was acquired using a SOMATOM Emotion single slice (Siemens Medical Systems, Erlangen, Germany) 80.0 mAs and 1500 ms exposure time, respectively. The distance between each slice was of 2.0 mm. The image was reconstructed on a matrix of 512 x 512 mm with pixels of 0.48 x 0.48 mm.

### Semi-quantification methods

Five semi-quantitative methods based on ROIs were applied by an experienced observer. The ROI-based quantitative methods were applied through semi-automated processes using (a) manually designed ROIs and (b) ROIs template and automated processes using (c) VOIs based on MR and CT structural images.

#### Manual ROI method

**Fig. 2:**
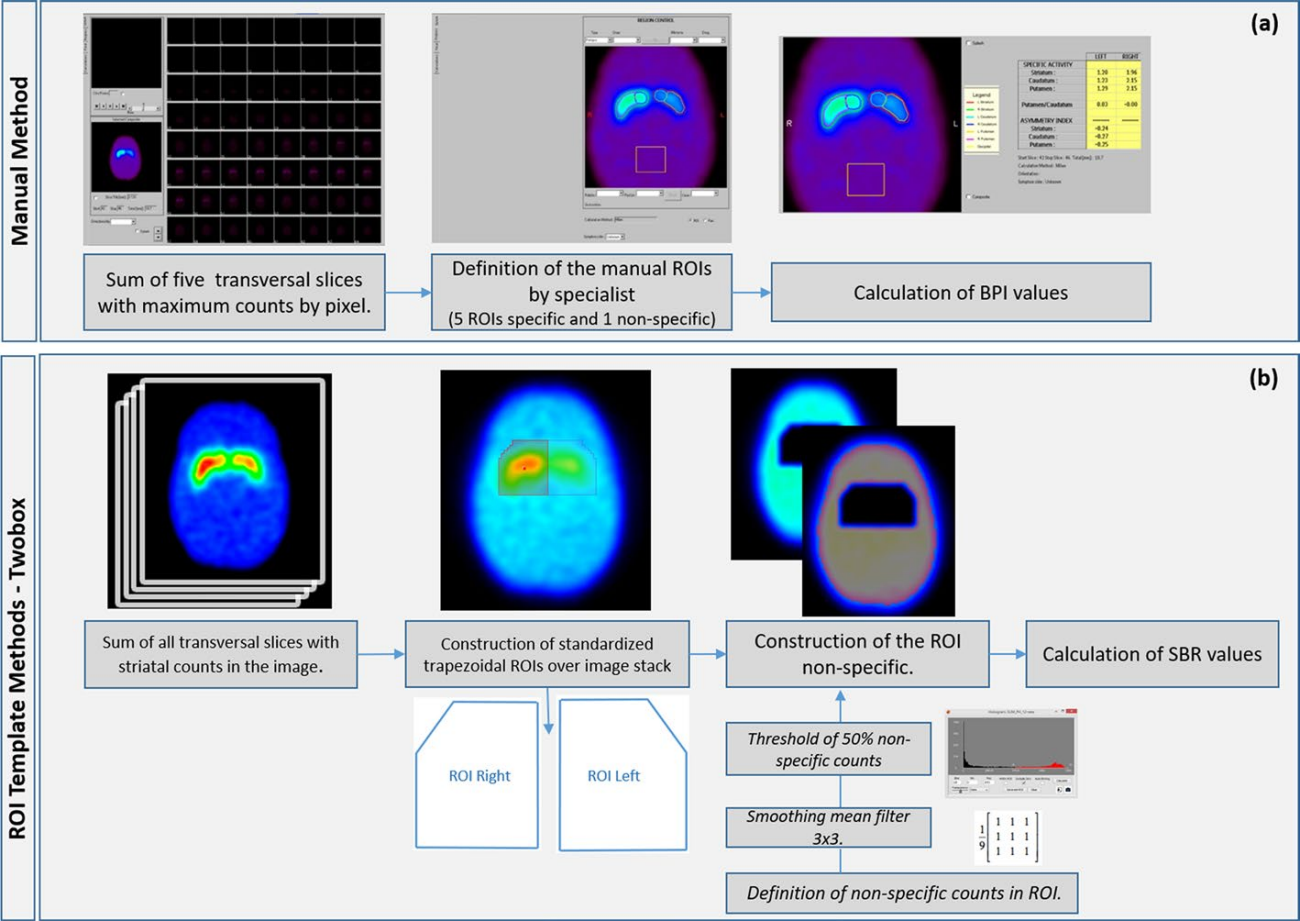
Pipeline to extraction and quantification to specific and non-specific counts in regions of interest in SPECT images through the (**a**) manual method and (**b**) template method—TwoBox

The study used the Brain Dopamine Transport (BDT) tool (EBW JetPack Philips, Philips Healthcare, Cleveland, Ohio, USA). It also selected five SPECT slices with the highest density of counts on the striatum, and that composed a quantitatively evaluated 2D image. An expert drew six specific ROIs manually on each compartment of interest—CAs, PUs, and STs. A reference ROI was constructed over the image’s region of low-density counts. BPI values for each compartment of the striatum were calculated using Eq. 1 [9] (Fig. 2a).

1 $$\begin{aligned} \textit{BPI}= \frac{C_{\mathrm{ROI(s)}} - C_{\mathrm{ROI(ns)}}}{C_{\mathrm{ROI(ns)}}} \end{aligned}$$

The equation, $C_{\mathrm{ROI(s)}}$ and $C_{\mathrm{ROI(ns)}}$ are average counts per pixel found in the respective specific (s) ROIs and non-specific (ns) reference ROI , respectively.

#### ROI Template Method

In the investigation of images of the ST, the quantification parameter defined as Specific Binding Ratio (SBR) (Eq. 2) is used in a manner analogous to the BPI correct the “spread” counts due to the partial volume effects (PVE) by a factor of weighting. The SBR is defined as the ratio of activity concentrations used to fill of the ST and the brain shell grooves.

2 $$\begin{aligned} \textit{SBR}= \frac{C_{\textrm{s}}}{C_{\textrm{ns}}} \end{aligned}$$

In the equation, $C_{\mathrm{s.}}$ corresponds to the concentration of counts exclusively originated from the compartments of the striatum, and $C_{\mathrm{ns.}}$ the concentration of counts from reference region.

The TwoBox method was based on the use of a standardized ROI template to collect quantification parameters in composite two-dimensional (CI-2D) SPECT images of the ST [12]. The main steps (Fig. 2b) involved were: (a) construction of a 2D image from the sum of cross-sections containing scored body counts; (b) positioning the pre-constructed trapezoidal ROIs over the striatal compartments on the 2D image, in order to ensure the recording of the density of counts present in each compartment and those resulting from the partial volume; (c) definition of the reference ROI for the calculation of the SBR semi-quantification parameter. The CI-2D images allow an analysis of ROIs by geometric VOI, taking into account the number of cross-sections used to form the 2D images. Cross sections with counts from ST compartments were selected by an expert so that they could be summed up in a single 2D image. In the construction of the standardized templates, two trapezoidal ROIs with standardized dimensions of approximately 44.8 x 38.4 mm were used on the 2D images. ROIs with large dimensions were used to detect the total density of counts of the ST grooves, including any signal from partial volume effects. In each 2D image, the experts positioned the ROIs symmetrically over the right and left ST. The construction of the reference ROI was semi-automated. After recording the total counts in each striatal ROI, an intrinsic binary mask was applied to preserve only the signal around the ROIs. Then, the resulting image containing only non-specific activity counts was smoothed out by a 3x3, 3-way filter applied three times to reduce the statistical fluctuations of the remaining signal in this reference region. Finally, a 50% threshold was set to delineate the reference ROI of each 2D image. SBR values were calculated in each phantom SPECT image using equation 3.

3 $$\begin{aligned} \textit{SBR}= \frac{C_{\mathrm{s.}}}{C_{\mathrm{ns.}}}=\frac{1}{V_{\textrm{ST}}} \cdot \left(\frac{Ct_{\mathrm{ROI(s)}}}{c_{\mathrm{ns.}}}-V_{\mathrm{ROI(s)}}\right) \end{aligned}$$

In the equation, $V_{\textrm{ST.}}$ is the known volume of each compartment of the striatum and $\hbox {V}_{\mathrm{ROI(s)}}$ is the volume of the ROI of interest used on the striatum. That is, the product of the area of the geometric ROI with the number of cross-sections used in the construction of the two-dimensional image, $\hbox {Ct}_{\mathrm{ROI(s)}}$ is the number of counts recorded by specific ROI and $\hbox {c}_{\mathrm{ns.}}$ the average number of counts recorded by the reference ROI.

The construction and application of the ThreeBox method was similar to the semi-automated technique described in the previous item, using similar standardized geometric ROIs. Also, the 2D images constructed from the sum of all cross-sections of the ST were used in this method. The quantification process involves the manual definition of two superficially equal rectangular ROIs constructed and positioned on the ST regions, to delimit the maximum density of counts. The reference ROI was constructed with the same area of the ST ROI and positioned just below it, allowing to the registration of a homogeneous distribution of the total counts. The ThreeBox method uses the Total Binding Potential Index (TBPI) as the semi-quantitative evaluation parameter (Eq. 4).

4 $$\begin{aligned} \textit{TBPI}= \frac{C_{\mathrm{s.}}}{C_{\mathrm{ns.}}}=\frac{1}{V_{\textrm{ST}}} \cdot \left(\frac{Ct_{\mathrm{ROI(s)}}-c_{\mathrm{ns}} \cdot V_{\mathrm{ns}}}{c_{\mathrm{ns}}\cdot V_{\textrm{ns}}}\right ) \end{aligned}$$

#### Automated Semi-quantification Methods

**Fig. 3:**
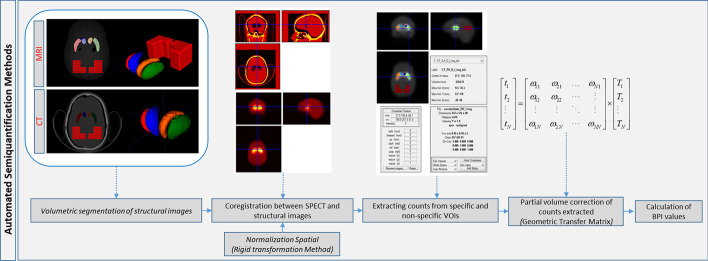
Pipeline to extraction and quantification to specific and non-specific counts in regions of interest in SPECT images through the automated semi-quantification methods

A fully automated method was developed and evaluated in the semi-quantification process of SPECT phantom images using the structural information of MRI and CT. The main steps involved were: (a) segmentation, volumetry and construction of a (VOI) for each striatal compartment in MRI and CT images, (b) co-registration between SPECT and structural images, (c) extraction of the values of the average counts per voxel recorded in each VOI, and finally (d) automated quantification of the BPI values already corrected for the partial volume effect (PVE) in each VOI analyzed. The initial segmentation stage was implemented on MRI and CT volumetric images, using the free software ITK-SNAP (www.ia.unc.edu/dev/download/index.html) [17]. The same process was used for MRI and CT images. VOIs were constructed for each ST compartment observed in both images. The reference VOIs were constructed in a posterior region of the brain shell and their volume were also recorded. SPECT images of the striatal phantom were co-registered with MRI and CT structural images using a rigid body transformation applied from the toolbox to MATLAB R2013.b (The MathWorks Inc., Natick, MA, USA), Statistical Parametric Mapping (SPM8) (Wellcome Department of Cognitive Neurology, London, UK). The mean counts in each compartment were then obtained with the aid of the VOIs constructed from the MRI and CT images. The space of the SPECT image was standardized for each structural image. Then, the MarsBar toolbox for SPM (MARSeille Boîte À Région d’Intérêt) was used to position each VOI in the CAs and PUs compartments in each SPECT image, automatically. Finally, the BPI indices found in each compartment were corrected for the PVE using the Geometric Transfer Matrix (GTM) method, making it possible to estimate the contribution of counts neighboring the volume of interest (Fig. 3). The coefficients GTM estimated are showing in Additional file 1.

### Statistical Analysis

Semi-quantitative indices (BPI, TBPI and SBR) were analyzed to evaluate the method’s performance. The Concordance Correlation Coefficient (CCC) was used to assess the accuracy of the indices. A linear association between the real and quantified values was evaluated using the Pearson coefficient (r) with the linear parameters estimated by the least squares model. Linearity parameters were evaluated using the Coefficient of Determination (COD) ($\rho ^{\textrm{2}}$). MedCalc v12.7 (MedCalc, Mariakerke, Belgium) and SPSS v18.0 (IBM Corporation, Armonk, NY, USA) were used in the analysis. The level of significance ($\alpha$) was 5% (two-tailed).

## Results

The mean actual values of BPI used for filling the striatal phantom according with each level of investigated activity are shown in Table 1. To avoid underestimating counts on the acquired SPECT images, all activity values recorded in this experiment were corrected for radioactive decay effects.

**Table 1:** Average proportions of the actual filling concentrations, simulating different concentrations of activity present in the compartments of the striatum

	Activity Concentration Real Levels(Mean ± SD)		
Filling Levels	High	Intermediary	Low
	(10:1 to 8:1)	(7:1 to 5:1)	(4:1 to 2:1)
BPI Real	9.6 ± 1.1	6.8 ± 1.1	3.7 ± 1.1

Before applying of the TwoBox and ThreeBox methods, the CI-2D images were constructed with the average sum of 19.78 ± 0.79 transversal images, resulting in an average relative thickness of 42.14 ± 1.79mm of the quantified images. The volumetry of the ST compartments of interest revealed the following MRI VOIs: CA(right) = 4.38 mL, CA(left) = 4.60 mL, PU (right) = 5.38 mL and PU(left) = 5.88 mL. The CT VOIs were: CA(right) = 4.67 mL, CA(left) = 4.59 mL, PU(right) = 5.62 mL and PU(left) = 6.18 mL. These VOIs involving structural information of MRI and CT presented a difference in volume below the spatial resolution of the SPECT images, with differences of up to 4.1% concerning the actual values of each compartment. The quantification indices used to evaluate the method’s reproducibility and accuracy are shown in Table 2.

**Table 2:** Semi-quantification results to any method applied on different structures of the striatum phantom. The indexes of quantification are represented for Binding Potential Index (BPI); Specific Binding Ratio (SBR) and Total Binding Potential Index (TBPI) in the quantification of cavities simulated of the caudate nucleus, putamen and striatum

Index of Quantification Measured to Different Levels Activity Concentration in Striatum Phantom(Mean ± SD)			
Method	High	Intermediary	Low
	(10:1 to 8:1)	(7:1 to 5:1)	(4:1 to 2:1)
Manual			
$\hbox {BPI}_{\textrm{CA}}$	3.70 ± 0.48	2.34 ± 0.32	1.09 ± 0.41
$\hbox {BPI}_{\textrm{PU}}$	3.57 ± 0.52	2.27 ± 0.41	1.00 ± 0.42
$\hbox {BPI}_{\textrm{ST}}$	3.31 ± 0.41	2.06 ± 0.34	0.93 ± 0.37
MRI Cor.			
$\hbox {BPI}_{\textrm{CA}}$	7.43 ± 0.92	4.49 ± 0.71	2.48 ± 0.83
$\hbox {BPI}_{\textrm{PU}}$	7.47 ± 0.93	4.87 ± 0.81	2.36 ± 0.85
$\hbox {BPI}_{\textrm{ST}}$	7.45 ± 0.91	4.90 ± 0.76	2.41 ± 0.84
CT Cor.			
$\hbox {BPI}_{\textrm{CA}}$	7.44 ± 0.93	4.84 ± 0.73	2.28 ± 0.86
$\hbox {BPI}_{\textrm{PU}}$	7.40 ± 0.92	4.76 ± 0.83	2.23 ± 0.87
$\hbox {BPI}_{\textrm{ST}}$	7.42 ± 0.91	4.80 ± 0.79	2.25 ± 0.86
TwoBox			
SBRST	8.52 ± 1.08	5.80 ± 0.83	2.99 ± 0.97
ThreeBox			
TBPIST(%)	102.29 ± 12.05	66.98 ± 9.68	31.53 ± 12.44

**Fig. 4:**
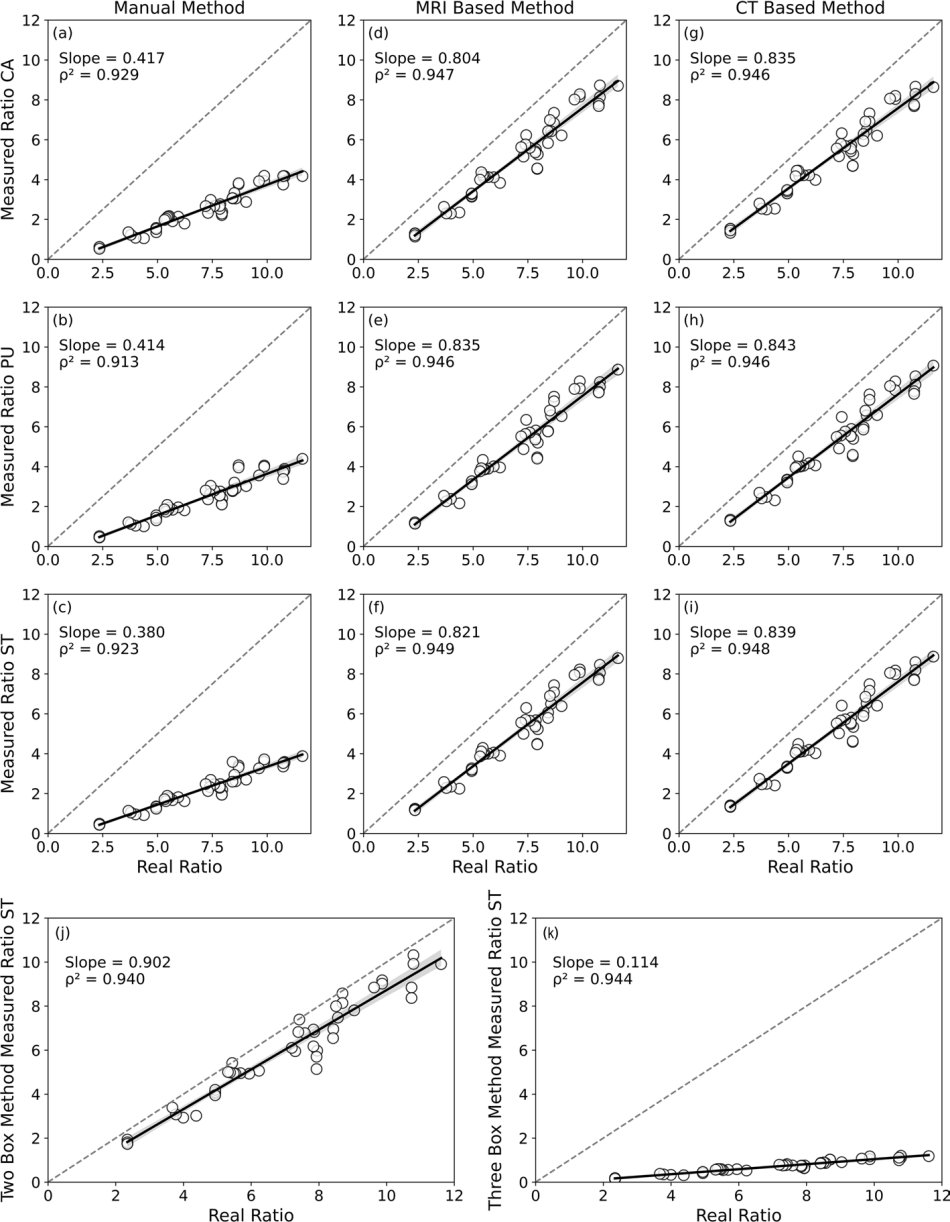
Linear relationship between the simulated uptake ratios and the measured indexes of each investigated structure to each quantification methodology assessed. The results striatum cavities measured by manual method (**a**–**c**), automatized methods (**d**–**i**) and semi-automatized methods (**j** and **k**) were compared and fitted with the real ratios fill through quality metrics to accuracy and precision to each quantitative method assessed

All evaluated methods had their results correlated with the actual activity rates used to fill each compartment, as shown in Table 3. Pearson’s coefficients were > 0.95 (p < 0.05) for all methods on each region of interest. The angular coefficients of each evaluated model presented values between 0.38 to 0.90, except for the ThreeBox method. The manual method showed values between 0.380 to 0.414, underestimating the real indices in > 50%. The Structural VOIs (i.e., MRI and CT) presented values between 0.80 to 0.84, and the TwoBox method showed a value of 0.9, close to the real indices. All linear models using each method’s corresponding variables and covariates showed an excellent fit ($\rho ^{\textrm{2}}$ > 0.91). Overall, the CCC values showed a low agreement between the actual filling values and the results measured by each method. The lowest CCC value was observed with the manual method (< 0.095). Otherwise, the best agreement occurred with evaluating of low activity levels by the TwoBox method (CCC = 0.75) (Fig. 4).

**Table 3:** Comparison of the performance parameters to quantitative methodsConcordance Correlation Coefficients (CCC) for the evaluation of the accuracy of each semiquantitative method, linear regression coefficients, Pearson coefficients ($\rho$) and Coefficient of Determination ($\rho ^{\textrm{2}}$)

Method	Index measured$^{1}$	Concordance correlation coefficient-CCC			Coefficients of linear regression analysis$^{2}$					
		High	Intermediary	Low						
		(10:1 to 8:1)	(7:1 to 6:1)	(4:1 to 2:1)	Intercept (CI)	SE	Slope (CI)	SE	$\rho$	$\rho ^{\textrm{2}}$
Manual										
	$\hbox {BPI}_{\textrm{CA}}$	0.021 (0.003 ; 0.039)	0.028 (0.007 ; 0.049)	0.095 (0.011 ; 0.177)	-0.435(-0.692;-0.178)	0.127	0.417 (0.38;0.452)	0.017	0.964	0.929
	$\hbox {BPI}_{\textrm{PU}}$	0.017 (-0.001 ; 0.035)	0.034 (0.009 ; 0.059)	0.096 (0.013 ; 0.177)	-0.504 (-0.789;-0.219)	0.141	0.414 (0.375;0.453)	0.019	0.959	0.913
	$\hbox {BPI}_{\textrm{ST}}$	0.013 (0.000 ; 0.026)	0.026 (0.007 ; 0.046)	0.081 (0.010 ; 0.152)	-0.457 (-0.700;-0.210)	0.121	0.38 (0.346;0.413)	0.016	0.961	0.923
MRI										
	$\hbox {BPI}_{\textrm{CA}}$	0.235 (0.072 ; 0.386)	0.255 (0.099 ; 0.399)	0.495 (0.221 ; 0.697)	-0.464(-0.885;-0.043)	0.208	0.804(0.747;0.861)	0.028	0.974	0.947
	$\hbox {BPI}_{\textrm{PU}}$	0.243 (0.073 ; 0.398)	0.265 (0.106 ; 0.411)	0.456 (0.189 ; 0.661)	-0.722(-1.164;-0.28)	0.219	0.835(0.775;0.895)	0.029	0.973	0.946
	$\hbox {BPI}_{\textrm{ST}}$	0.239 (0.074 ; 0.391)	0.261 (0.104 ; 0.405)	0.473 (0.203 ; 0.677)	-0.608(-1.033;-0.182)	0.211	0.821(0.763;0.879)	0.028	0.974	0.949
CT										
	$\hbox {BPI}_{\textrm{CA}}$	0.239 (0.072 ; 0.392)	0.244 (0.093 ; 0.383)	0.438 (0.177 ; 0.642)	-0.766(-1.21;-0.322)	0.22	0.835(0.775;0.896)	0.029	0.973	0.946
	$\hbox {BPI}_{\textrm{PU}}$	0.226 (0.065 ; 0.376)	0.252 (0.100 ; 0.393)	0.421(0.163 ; 0.625)	-0.878(-1.326;-0.43)	0.222	0.843(0.782;0.903)	0.03	0.973	0.946
	$\hbox {BPI}_{\textrm{ST}}$	0.233 (0.071 ; 0.383)	0.248 (0.097 ; 0.388)	0.428 (0.169 ; 0.632)	-0.828(-1266;-0.391)	0.217	0.839(0.78;0.899)	0.029	0.974	0.948
TwoBox										
	SBR	0.535 (0.242 ; 0.738)	0.497 (0.241 ; 0.688)	0.750 (0.488 ; 0.888)	-0.293(-0.8;0.213)	0.251	0.902(0.833;0.971)	0.034	0.969	0.94
ThreeBox										
	TBPI	0.002(0.000 ; 0.004)	0.005(0.004 ; 0.008)	0.027 (0.002 ; 0.053)	-4.467(-5.303;-3.633)	0.41	0.114(1.31;1.552)	0.056	0.972	0.944

$^{1}$ The indexes are represented through of the quality parameter and confidence interval.

$^{2}$ The coefficients are represented with the confidence interval (CI) and standard error(SE)

## Discussion

This study evaluated the performance (accuracy and precision) of five methods developed for semi-quantification of SPECT images under controlled conditions using a striatal anthropomorphic phantom. The methods were based on ROIs manually constructed and positioned, standardized templates and the aid of volumetric information provided by CT and MRI images. The semi-quantitative results of each method were compared with the actual filling values used in the simulator. The activity concentrations used in each phantom compartment were defined to observe similar results to those found in the clinical evaluation of normal and degraded patterns of striatal uptake. DAT semi-quantification methods of SPECT images are important due to the applicability of these routines in image processing by algorithms of normalization and segmentation [17]. Pearson’s coefficient values highlighted the excellent linear correlation between the semi-quantitative results and the actual filling values for all methods investigated, similar to evaluations using $^{\textrm{123}}$I [18], as well as, between the quantification values and the expected results was observed. On the other hand, the CCC was adopted as an accuracy metric of the semiquantitative methods. Overall, all methods demonstrated a greater accuracy during the identification of lower concentration of activity, regardless of the compartment investigated. However, all CCC values were low, indicating relative quantification deficiencies. When evaluating the complete striatum, the best accuracy was observed with the TwoBox method (CCC = 0.75, for low activity levels). For the evaluation of smaller structures such as CA and PU, only MRI (0.49 and 0.46) and CT (0,44 and 0,42) showed superior performance for lower activity concentrations. The interference of partial volume effects was observe in the quantification of data. The angular coefficients found with the manual method in the different grooves of the striatum (0.38 to 0.42) show that the counts measured in each ROI manually constructed and positioned were underestimated. The counts not recorded by the manual ROIs resulted in underestimated quantified indices of at least 60% compared to the actual data used in the padding. It is critical to how the subjectivity of the operator interferes in different stages of the quantification process. Implementing of automated or semi-automated resources is indispensable for minimizing the biases inserted into the quantification indices [19]. Therefore, partial volume correction (PVC), applied through the GTM method, is an indispensable resource in quantifying striatal SPECT images, as evidenced by the angular coefficients of the structural methods close to one (MRI VOIs = 0.80 to 0.83 and CT VOIs = 0.83 to 0.84). The result of the co-registration of the structural images with the SPECT images using a rigid body transformation can be evaluated visually by verifying the overlap of each VOI on their respective quantification sites. In a clinical scenario, semi-quantification methodologies using the standardization of the structural images for the MNI (Montreal Neurological Institute) space prior to the co-registration with the SPECT images of the striatum suggest similar results to that observed in this study [20], although extrastriatal uptake levels can interfere with the performance of spatial normalization algorithms, especially in cases of patients with disorders of dopaminergic transporters and receptors. The TwoBox method showed semi-quantification results closer to the values present in the striatal simulator. Its excellent performance in evaluating and identifying values close to the real ones is related to the capacity of collecting all the counting density from the compartments of interest by the geometrically large ROIs, although when applied in clinical cases, this measurements possibly are committed due the contamination by low uptake areas outside the brain than content cerebrospinal fluid such as sulci and ventricles [21, 22] . Moreover, it uses the ratio between the volumes of the striatum and the relative volume of the built geometric ROI as a weighting factor. The TwoBox method was evaluated in different clinical conditions of uptake [10, 15, 23], but in few studies with a striatal phantom using ^99m^Tc as the isotope of interest. All semi-quantitative methods evaluated had more than 90% (COD) of their points suitable to their respective constructed linear models. Some limitations were presented in this study. The first limitation denotes the fact that only dopaminergic simulation images were used to evaluate the quantitative methods, and DAT SPECT images of patients with dopaminergic transporter disorders were not employed. In these cases, the residual activity due to low density of dopaminergic transporters may generate high density clusters of extra striatal counts and impair the performance of automated methods as used affine normalization techniques, which in turn reduce their efficiency in convoluting the images to a standard reference space. The second limitation of this study will be explored later is the performance of intra- and inter-rater reproducibility, since methods that require a delineation of ROIs by the rater may offer quantitative assessment parameters that are more sensitive to variation, to the point of impairing diagnostic information and follow-up response.

## Conclusion

This study evaluated the accuracy and precision of five methods for semi-quantification of the striatum using an anthropomorphic striatal phantom. The binding indices evidenced by each technique—manual, standard and semi-automated templates—were expressively different when the images were acquired under the same conditions. The subjectivity intrinsic to the observer related to the positioning, size and geometry of ROIs were the main factors responsible for the variability between the different results found. Likewise, the PVE contributed significantly to the underestimation of the quantified indices compared to the actual filling values and it is indispensable to correct them to ensure more accurate investigations. The semi-automated techniques with MRI and CT, when corrected for PVE, were those that presented a better accuracy for quantifying individual structures, such as CA and PU. However, the TwoBox technique showed the best performance in evaluating the binding rates of the striatum as a whole. All methodologies investigated showed an excellent co-linearity among their quantification indices.

## Supplementary Information

**Additional file 1: Table S1.** Geometric Transfer Matrix to the compartments of MRI image.** Table S2.** Geometric Transfer Matrix to the compartments of CT image

## Data Availability

All data are available under request.

## Abbreviations

BPI: Binding potential index
CA: Caudate nucleus
CCC: Concordance correlation coefficients
COD: Coefficient of Determination
CT: Computed tomography
DAT: Dopamine transporters
GTM: Geometric transfer matrix
LEHR: Low energy high resolution
MRI: Magnetic resonance imaging
ns: Non-specific
OSEM: Ordered subset expectation maximization
PU: Putamen
PVE: Partial volume effects
ROI: Region of interest
s: Specific
SBR: Specific binding ratio
SPECT: Single photon emission computed tomography
ST: Striatum
TBPI: Total binding potential index
VOI: Volume of interest
