# Supplementary material for: Assessment of different regions of interest-based methods for [99mTc]Tc DAT-SPECT quantification using an anthropomorphic striatal phantom
Source: EJNMMI Phys. 2022 Dec 28;9:91. doi: 10.1186/s40658-022-00519-2 (PMC9797635; doi:10.1186/s40658-022-00519-2)
Supplement: Supplementary file 1 — Additional file 1: Table S1. Geometric Transfer Matrix to the compartments of MRI image. Table S2. Geometric Transfer Matrix to the compartments of CT image [file 40658_2022_519_MOESM1_ESM.pdf]

Table 1 - Geometric Transfer Matrix to the compartments of MRI image.

	Correction factor by compartment of MRI				
**Compartment Corrected**	**Caudate Right**	**Putamen Right**	**Caudate Left**	**Putamen Left**	**Reference Region**
Caudate Right	2.75	0.30	-0.04	0.00	-1.06
Putamen Right	-0.25	2.41	0.00	0.00	-0.91
Caudate Left	-0.06	0.00	2.72	-0.36	-0.98
Putamen Left	0.00	0.00	-0.27	2.33	-0.82
Reference Region	0.00	0.00	0.00	0.00	1.00

Table 2 - Geometric Transfer Matrix to the compartments of CT image.

	Correction factor by compartment of MRI				
**Compartment Corrected**	**Caudate Right**	**Putamen Right**	**Caudate Left**	**Putamen Left**	**Reference Region**
Caudate Right	2.81	-0.29	-0.05	0.00	-1.38
Putamen Right	-0.24	2.42	0.00	0.00	-1.13
Caudate Left	-0.04	0.00	2.78	-0.34	-1.33
Putamen Left	0.00	0.00	-0.26	2.38	-1.05
Reference Region	0.00	0.00	0.00	0.00	1.00

.
